# A complete graph-based approach with multi-task learning for predicting synergistic drug combinations

**DOI:** 10.1093/bioinformatics/btad351

**Published:** 2023-06-01

**Authors:** Xiaowen Wang, Hongming Zhu, Danyi Chen, Yongsheng Yu, Qi Liu, Qin Liu

**Affiliations:** School of Software Engineering, Tongji University, Shanghai 201804, China; School of Software Engineering, Tongji University, Shanghai 201804, China; School of Software Engineering, Tongji University, Shanghai 201804, China; Drug Discovery and Development Department, Tasly Biopharmaceuticals Co., Ltd, Shanghai 201203, China; Translational Medical Center for Stem Cell Therapy and Institute for Regenerative Medicine, Shanghai East Hospital, Bioinformatics Department, School of Life Sciences and Technology, Tongji University, Shanghai 200092, China; School of Software Engineering, Tongji University, Shanghai 201804, China

## Abstract

**Motivation:**

Drug combination therapy shows significant advantages over monotherapy in cancer treatment. Since the combinational space is difficult to be traversed experimentally, identifying novel synergistic drug combinations based on computational methods has become a powerful tool for pre-screening. Among them, methods based on deep learning have far outperformed other methods. However, most deep learning-based methods are unstable and will give inconsistent predictions even by simply changing the input order of drugs. In addition, the insufficient experimental data of drug combination screening limits the generalization ability of existing models. These problems prevent the deep learning-based models from being in service.

**Results:**

In this article, we propose CGMS to address the above problems. CGMS models a drug combination and a cell line as a heterogeneous complete graph, and generates the whole-graph embedding to characterize their interaction by leveraging the heterogeneous graph attention network. Based on the whole-graph embedding, CGMS can make a stable, order-independent prediction. To enhance the generalization ability of CGMS, we apply the multi-task learning technique to train the model on drug synergy prediction task and drug sensitivity prediction task simultaneously. We compare CGMS’s generalization ability with six state-of-the-art methods on a public dataset, and CGMS significantly outperforms other methods in the leave-drug combination-out scenario, as well as in the leave-cell line-out and leave-drug-out scenarios. We further present the benefit of eliminating the order dependency and the discrimination power of whole-graph embeddings, interpret the rationality of the attention mechanism, and verify the contribution of multi-task learning.

**Availability and implementation:**

The code of CGMS is available via https://github.com/TOJSSE-iData/CGMS.

## 1 Introduction

Drug combination therapy is considered to be the cornerstone of cancer treatment (Mokhtari *et al.* 2017; Fudio *et al.* 2022). At present, more than 350 drug combinations have been clinically used for cancer treatment (Fudio *et al.* 2022). Compared with monotherapy, the co-involvement of two or more drugs is expected to inhibit multiple oncogenic pathways simultaneously (Gilad *et al.* 2021), resulting in higher efficacy, more delayed drug resistance and lower toxicity (Mokhtari *et al.* 2017). However, studies looking for synergistic drug combinations suffer from substantial failure rates (Fudio *et al.* 2022). Many synergistic drug combinations are proposed based on clinical experience and *in vivo* experiments, which are time-consuming, labor-intensive, and may expose patients to harmful treatments (Wang *et al.* 2022b). Although high-throughput screening (HTS) technology can screen drug combinations *in vitro*, the technology is expensive to build (Macarron *et al.* 2011) and cannot test the full combinational space. In order to discover anti-cancer synergistic drug combinations more efficiently and at low cost, researchers started to apply computational methods to predict synergistic drug combinations for pre-screening.

Early computational models were usually only applicable to specific targets, pathways, diseases, or cell lines (Preuer *et al.* 2018). In the past decade, researchers have started to utilize machine learning-based methods (Li *et al.* 2015; Ding *et al.* 2019; Sidorov *et al.* 2019) to predict synergistic drug combinations with omics data, medicinal chemical structures, or biological networks. With the increasing accumulation of experimental data (O’Neil *et al.* 2016) from techniques such as HTS, deep learning (DL) began to be involved for drug combination prediction. DeepSynergy (Preuer *et al.* 2018) is the earliest research that applies DL to predict synergistic drug combinations, which predicts the Loewe Synergy Score (Loewe 1953) of drug combinations with the multi-layer perceptron (MLP) and achieves far better performance than classical ML methods. AuDNNSynergy (Zhang *et al.* 2021) improves the prediction precision on the basis of DeepSynergy by introducing auto-encoder for feature dimensionality reduction. Afterwards, the maturity of graph neural networks (GNNs) further inspired researchers. PRODeepSyn (Wang *et al*. 2022b) utilizes the graph convolutional network (GCN) model to construct more predictive cell line features to predict synergistic drug combinations, while DeepDDS (Wang *et al.* 2022a) utilizes GNNs to construct more informative drug features for predictions. Jiang *et al.* (2020) utilizes the GCN model to predict synergism between drug nodes based on biomedical networks. GraphSynergy (Yang *et al.* 2021) proposed a GCN model with attention mechanism to predict synergistic drug combinations. TranSynergy (Liu and Xie 2021) predicts drug combinations through the Transformer model (Vaswani *et al.* 2017) which also has a powerful attention mechanism. There are also DL-based studies that predict synergistic drug combinations with tensor factorization (DTF) (Sun *et al.* 2020) or deep cascade forest (EC-DFR) (Lin *et al.* 2022).

Although the rise of DL techniques has injected new impetus into predicting anti-cancer synergistic drug combinations, these DL-based models still have a long way from being in service. For example, most of the DL-based models are unstable and will give inconsistent predictions even if just changing the input order of drugs from (*Drug A*, *Drug B*, *Cell line C*) to (*Drug B*, *Drug A*, *Cell line C*). Despite the fact that many studies try to force models to give order-independent predictions through data augmentation (Preuer *et al.* 2018; Zhang *et al.* 2021; Wang *et al.* 2022b), models are still unable to truly reveal the association between drug combinations and cell lines (Atarashi *et al.* 2017). We have noticed that GraphSynergy has implemented the order-independent deep feature generation, however, it re-introduces the issue about order when making the final prediction.

Another factor hindering the application of DL-based models is that the generalization ability of existing models is still unsatisfactory. DL is a data-driven technology. Although many synergy datasets such as O’Neil dataset (O’Neil *et al.* 2016) and NCI-ALMANAC (Holbeck *et al.* 2017) have been published, the amount of published drug synergy datasets is still far less than the possible combination of drugs and cell lines. Furthermore, the number of synergistic drug combinations is much smaller than the number of non-synergistic ones, which seriously affects the precision and generalization ability of DL models. Existing studies have adopted techniques such as multi-model ensemble (Ding *et al.* 2019) and transfer learning (Kim *et al.* 2021) to obtain more accurate prediction results. However, multi-model ensemble is costly, and transfer learning still relies on large-scale pre-training data.

In this article, we propose a Complete Graph-based approach with Multi-task learning to identify anti-cancer Synergistic drug combinations named CGMS. A complete graph is a graph such that each pair of different nodes in the graph is connected with one and only one edge. CGMS regards a drug combination and a cell line as a heterogeneous complete graph, where two drug nodes and a cell line node are interconnected, to learn the relation between them. Then CGMS generates order-independent whole-graph embeddings for predicting synergy scores by leveraging heterogeneous graph attention network (HAN) (Wang *et al.* 2019). Meanwhile, the attention mechanism in HAN could encourage the model to focus more on the important parts in different samples adaptively, making the model generate more discriminative whole-graph embeddings. To improve the generalization ability of the model, we adopt the multi-task learning technique to train CGMS. We select the prediction of drug synergy scores and drug sensitivity scores as two learning tasks, since the calculation of synergy score is related to the sensitivity of the cell line to each drug in the combination. By learning more common knowledge from multiple tasks, the model can avoid overfitting a certain task, thereby improving the generalization ability (Zhang and Yang 2021). We evaluate CGMS on a widely used public dataset in three different scenarios, including the leave-drug combination-out scenario, the leave-cell line-out scenario, and the leave-drug-out scenario. CGMS outperforms the state-of-the-art methods in all scenarios. We also present the benefit of eliminating the impact of order dependency on the synergy predictions and the discrimination power of whole-graph embeddings. We analyze the interpretability of CGMS’s attention mechanism as well. Besides, we verify the improvement brought by applying multi-task learning. Overall, CGMS provides a possible direction for anti-cancer synergistic drug combination prediction models to move towards application.

## 2 Materials and methods

### 2.1 Datasets

We used the large-scale combination screening data published by O’Neil *et al.* (2016) to evaluate CGMS. The experimental data covers the *in vitro* testing results of 583 different drug combinations against 39 human cancer cell lines. Referring to Preuer *et al.* (2018), we constructed the synergy dataset which consists of 22 737 samples and their Loewe scores. We also constructed the sensitivity dataset based on DrugComb (Zagidullin *et al.* 2019) to train CGMS with multi-task learning. We select the relative inhibition (RI) value as the sensitivity score of a single drug, since the RI value is more robust than other methods in characterizing drug sensitivity (Douglass *et al.* 2022) and can be used to compare drug response in different concentration ranges (Zagidullin *et al.* 2019). We finally constructed a sensitivity dataset of 11 766 samples with 346 drugs and 175 cell lines for CGMS. Please see Supplementary Material S1 for more details.

### 2.2 Drug features

The molecular fingerprints and descriptors were used to construct drug feature vectors to retain the information on the drug structures and physicochemical properties of the drugs. We collected the simplified molecular input line entry system (SMILES) expressions of 3118 drugs from DrugComb and utilized the RDKit toolkit (Landrum *et al.* 2013) to extract the fingerprints and molecular descriptors of each drug. We calculated the Morgan fingerprint with the radius of 3 and converted the fingerprint into a 1024-dimensional binary vector. We also calculated 189 non-zero variance molecular descriptors for the collected drugs. Then the drug fingerprint vectors and molecular descriptors were concatenated and *z*-score normalized to form the original drug feature matrix. Finally, we used auto-encoder to encode drug features to 256 dimension (see Supplementary Material S2).

### 2.3 Cell line features

The cell line features were mainly constructed based on the RNA-Seq transcript per million (TPM) gene expression data provided by Cancer Cell Line Encyclopedia (CCLE) (Barretina *et al.* 2012), in which the TPM value has been processed by logarithm. Here we obtained expression data from CCLE for 19 177 genes of 174 cell lines. Since the gene expression data of cell line *OCUB-M* was unable to be obtained from CCLE, we supplemented the TPM data of *OCUB-M* from Cell Model Passports (van der Meer *et al.* 2018). After that, we screened out genes with the largest variance of gene expression level among 175 cell lines and kept 5001 genes which may carry more information. The gene expression data was also preprocessed with *z*-score normalization and encoded as 256-dimensional vectors (see Supplementary Material S2).

### 2.4 CGMS

#### 2.4.1 The heterogeneous complete graph

**Figure 1:**
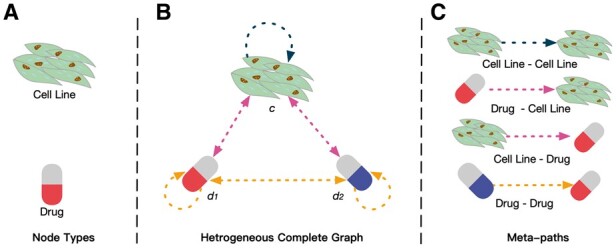
The heterogeneous complete graph for drug combinations and cell lines. (A) Two node types in the complete graph. (B) The heterogeneous complete graph with three nodes and six edges. (C) Four types of meta-paths in the complete graph.

CGMS is a complete graph-based approach for anti-cancer synergistic drug combination prediction trained with multi-task learning. In this article, a heterogeneous complete graph is built to describe the interaction between drug combinations and cell lines. As presented in Fig. 1, the constructed graph contains nodes for both cell line and drug types. Each node with a self-loop is connected by binary-directed edges. There are four types of possible meta-paths as illustrated in Fig. 1C. The paths drug–cell line and cell line–drug are used to simulate the therapeutic effect of a single drug on the cell line, and the binary-directed edge reflects that the efficacy depends on both the drug and the cell line. The path drug–drug reflects that drugs may interact due to the chemical structure and other factors. The existence of self-loops can help to extract drug and cell line features without forgetting the information of the nodes themselves (Jin *et al.* 2021). Afterwards, CGMS can generate the order-independent whole-graph embedding to represent the drug combination and the cell line for later prediction.

#### 2.4.2 Generation of the whole-graph embedding

**Figure 2:**
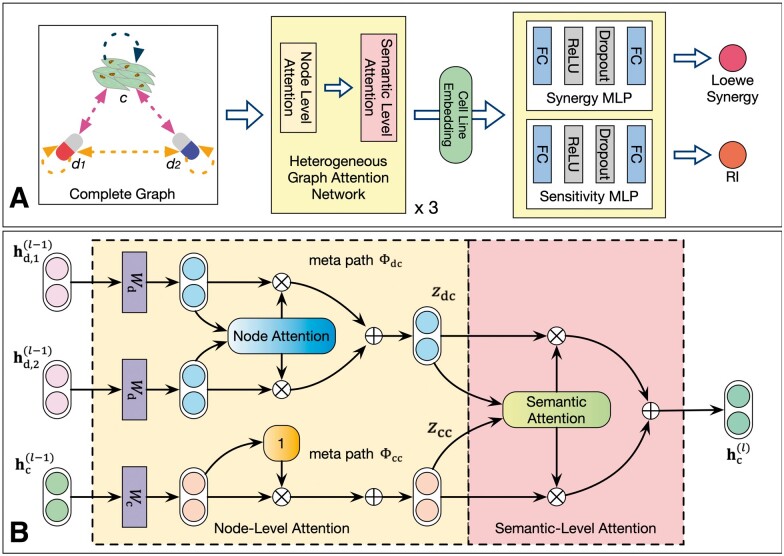
Workflow of CGMS. (A) The whole-graph embedding is generated with HAN and fed into two MLPs to predict the Loewe synergy score and the RI value, respectively. (B) The generation of the cell line embedding in layer *l* of HAN. The features of each node will first be projected to the same feature space and then aggregated through node-level attention depending on the meta-path. Finally, the cell line embedding is generated through semantic-level attention.

As shown in Fig. 2A, for the constructed complete graph, we use the heterogeneous graph attention network (HAN) (Wang *et al.* 2019) with three layers to generate the whole-graph embedding, which is the cell line node embedding output by the third layer of HAN. As shown in the figure, a HAN layer follows a hierarchical attention structure: from the node-level attention to the semantic-level attention. The self-attention mechanism is used to adaptively control the flow of information between nodes to reflect the importance of each node to the final synergy score, which makes the model more expressive.

Figure 2B shows the generation process of the cell line embedding in one layer of HAN. First of all, the features of each node will be projected to the same feature space with type-specified projection matrix *W*. In layer *l*, the representation **h** of node *u* with type ϕ is transformed as


(1) $$h_{u*}^{\left(l\right)}=W_{\phi }\cdot h_{u}^{\left(l-1\right)},\;u\in \phi$$

After projection, the heterogeneous graph could be regarded as a homogeneous graph. Afterwards, HAN performs node-level aggregation on nodes accessible through the same meta-path for each node. Given a meta-path Φ, the set of neighbors reachable by node *u* via Φ is denoted as $N_{\left(\Phi ,u\right)}$. The attention of node *u* to node *v* in $N_{\left(\Phi ,u\right)}$ is where $a_{\Phi }^{T}$ is the attention vector related to the meta-path, *σ* is the leaky rectified linear unit activate function, and [·||·] represents vector concatenation. In CGMS, since there is always one and only one cell line node in the complete graph, the attention of each node to the cell line node is always 1 when calculating the node-level attention. Through node-level attention, we can get the feature representation of node u under meta-path Φ as


(2) $$e_{uv}^{\Phi }=\text{exp}(\sigma (a_{\Phi }^{T}\cdot [h_{u*}||h_{v*}]))$$

(3) $$\alpha_{uv}^{\Phi }=\frac{e_{uv}^{\Phi }}{\sum_{k\in N_{\left(\Phi ,u\right)}}e_{uk}^{\Phi }}$$

(4) $$z_{u}^{\Phi }=\sigma \left(\sum_{v\in N_{\left(\Phi ,u\right)}}\alpha_{uv}^{\Phi }\cdot h_{v*}\right)$$

Similarly, the semantic-level attention is calculated to aggregate the representations of the same node under different meta-paths. The attention weights, or the importance, of each meta-path $\left\{\Phi_{0},\Phi_{1},\ldots ,\Phi_{m}\right\}$ related to node *u* is calculated as where q is the semantic-level attention vector. Finally, the representation output of node *u* in the *l*th layer is


(5) $$w_{u}^{\Phi_{i}}=\text{exp}(q^{T}\cdot \tanh(W\cdot z_{u}^{\Phi_{i}}+b))$$

(6) $$\beta_{\Phi_{i}}=\frac{w_{u}^{\Phi_{i}}}{\sum_{j=1}^{m}w_{u}^{\Phi_{j}}}$$

(7) $$h_{u}^{\left(l\right)}=\sum_{i=1}^{m}\beta_{\Phi_{i}}\cdot z_{u}^{\Phi_{i}}$$

In addition, we also adopt the multi-head attention mechanism to obtain more stable feature representations. For a given number of heads *N*, the feature representation of node *u* under meta-path $\Phi_{i}$ will be calculated *N* times under different parameters, and the resulting vectors will be sequentially concatenated as $z_{u}^{\Phi_{i}}$ to calculate the semantic-level attention.

As can be seen from the above formulas, the representation output of each node in the complete graph is the weighted sum of all nodes’ projected features, where the weights are calculated with the self-attention mechanism. Therefore, the whole-graph embedding is independent of the input order of drugs.

#### 2.4.3 Prediction for multiple tasks

To enhance the generalization ability of the model, we apply multi-task learning (Zhang and Yang 2021) to train the model. The chosen training tasks include the synergy score prediction for drug combinations and the sensitivity score prediction for a single drug, both of which use the mean square error (MSE) as the loss function. The synergy score is the difference between the observed efficacy and the expected efficacy of drug combinations, and the calculation of expected efficacy requires drug response of each drug (Loewe 1953). The RI value used to characterize drug sensitivity is calculated based on the area under single drug’s dose–response curve (Zagidullin *et al.* 2019). Therefore, we think that the two prediction tasks are related, from which CGMS could learn more general knowledge of the drug effect on cell lines. As shown in Fig. 2A, the whole-graph embedding of the complete graph is regarded as the general knowledge and accepted by two separate MLPs for different tasks. Each MLP has one hidden layer whose number of neural units is the half of the whole-graph embedding’s dimension, followed by a rectified linear unit activation layer and a dropout layer. It is worth pointing out that for the sensitivity score prediction task, we set the feature of the same drug as the node feature for two drug nodes as input, so that the obtained whole-graph embedding can represent the interaction between one single drug and one cell line.

Considering the inconsistent volume of drug sensitivity data and synergy data, we propose a modified joint training strategy to train the CGMS model. At each training step, we will randomly sample a batch of data for only one task, and accumulate the gradients in continuous steps. Only after the model is trained on the synergy prediction task, will we propagate back gradients to update the model parameters. Due to the smaller volume of the sensitivity dataset, we allow repeated sampling of the drug sensitivity data and control the non-replacement sampling of the synergy dataset. One epoch ends after all training samples from the synergy dataset have been trained on once.

## 3 Results

### 3.1 Experimental setup

We compared CGMS with six state-of-the-art methods, including one ensemble method named EC-DFR; two pure MLP-based methods, named DeepSynergy and AuDNNSynergy; two GCN-based methods, named PRODeepSyn and DeepDDS; and one transformer-based method named TranSynergy. We evaluated the generalization ability of CGMS and other methods in three different scenarios using 5-fold cross-validation. In the leave-drug combination-out scenario, the synergy dataset was evenly divided into five folds where drug combinations contained in one fold never occurred in the other folds. The sensitivity dataset was evenly divided to make all drugs occur in each fold, which was consistent with the synergy dataset. All the methods were trained on four folds and tested on the leave-out fold iteratively. Similarly, in the leave-cell line-out scenario or the leave-drug-out scenario, both the two datasets were divided into five folds to guarantee that the cell lines or drugs in the test fold were isolated from the other folds, respectively. The details of dataset division are described in Supplementary Material S1. With the three scenarios, we could evaluate the generalization ability of methods when facing novel drug combinations, novel cell lines, or novel drugs.

We treat the identification of anti-cancer synergistic drug combinations as a regression task, considering that treating it as a classification problem could oversimplify the reality (Preuer *et al.* 2018). We evaluated all methods mainly by calculating the MSE based on the predicted Loewe synergy scores and the ground truth. We also report the root MSE (RMSE) and the Pearson correlation coefficient (PCC). We re-implemented the methods that did not provide related metrics in paper following their original settings as possible as we could (Supplementary Material S3). We used nested cross-validation to select hyperparameters for CGMS. The learning rate was chosen in the set of {1e-3, 1e-4, 1e-5}, and the hidden layer size of the graph attention network was chosen in the set of {128, 256, 512}. We fixed the number of attention heads to 8 and the batch size to 512. The dropout rate was set to 0.5. The optimizer used was the AdamW optimizer. The maximum number of epochs per training was 500. We also adopted the Early Stopping technique to further avoid overfitting. See Supplementary Material S4 for more details.

### 3.2 Method comparison

The comparison was carried out in three different scenarios. We first evaluated CGMS and the selected baseline methods in the leave-drug combination-out scenario. The results are shown in Table 1. CGMS achieves the lowest MSE of 208.38 and the highest PCC of 0.78 among all compared methods. It reduces the MSE by 9.2% compared to the second best, GCN-based method PRODeepSyn. The performance of CGMS and PRODeepSyn indicates that graph-based methods are more likely to achieve better performance than pure MLP-based methods. Compared to TranSynergy that also has a powerful attention mechanism, CGMS achieves a 9.8% lower MSE and 0.03 higher PCC on a larger dataset, which we attribute to CGMS’s elimination of the impact of input order and the introduction of multi-task learning. The results of DeepDDS and EC-DFR are less competitive, which may be owning to the inappropriate scenarios (Supplementary Material S3). We further evaluated CGMS and five other methods in the more challenging scenarios. In the leave-cell line-out or leave-drug-out scenario, methods are required to predict the synergy scores for novel cell lines or novel drugs, which can better test the generalization ability of methods. Although all methods perform obviously worse than in the leave-drug combination-out scenario, CGMS still achieves the lowest MSE in both scenarios (Supplementary Tables S1 and S2). In summary, the results show that CGMS has significantly better generalization ability and outperforms other advanced methods when predicting the synergy scores of drug combinations.

**Table 1.** Results of method comparison on the synergy score prediction task.

Method	MSE	RMSE	Confidence interval 95%	PCC
CGMS	**208.38 ± 36.16**	**14.38 ± 1.23**	**[163.48, 253.28]**	**0.78 ± 0.02**
PRODeepSyn	229.49 ± 42.81	15.09 ± 1.37	[176.34, 282.64]	0.75 ± 0.02_5_
TranSynergy	231 ± 21			0.75 ± 0.02
AudnnSynergy	241.12 ± 43.52	15.46 ± 1.44	[187.09, 295.15]	0.74 ± 0.03
DeepSynergy	255.49	15.91 ± 1.56		0.73 ± 0.04
DeepDDS	271.62 ± 30.15	16.45 ± 0.93	[234.18, 309.05]	0.38 ± 0.06
EC-DFR	314.26 ± 57.09	17.66 ± 1.57	[243.38, 385.13]	0.62 ± 0.02_5_

*Note*: Values of MSE, RMSE, and PCC are mean values ± 1 standard deviation. The best results are shown in bold.

### 3.3 Predictions with different input orders of drug pairs

**Figure 3:**
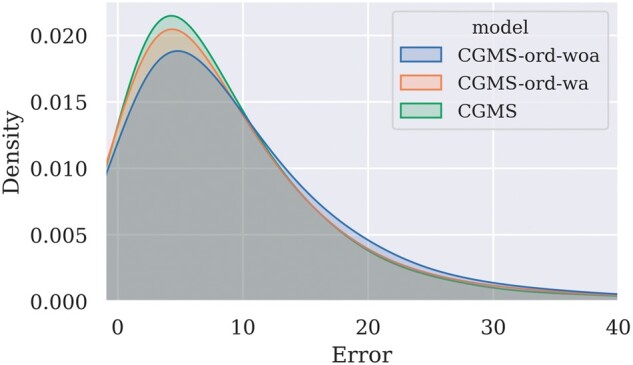
KDE plot on absolute errors between the predictions and the ground truth.

CGMS is designed to learn the order-independent embedding of a drug combination and a cell line. To illustrate the impact of the order dependency on the synergy prediction results of CGMS and other GCN-based methods, we evaluated CGMS, GraphSynergy (Yang *et al.* 2021), and DeepDDS with their variants in the leave-drug combination-out scenario. Specifically, for CGMS, we implemented CGMS-ord that replaced the whole-graph embedding with the concatenation of node embeddings to make predictions. Due to the concatenation, CGMS-ord became order dependent. For GraphSynergy and DeepDDS, we utilized the self-attention mechanism to implement the order-independent variants named GraphSynergy-indep and DeepDDS-indep, respectively. We also introduced the MLP structure into GraphSynergy for a more fair comparison. The implementation details are described in Supplementary Material S6. Each order-dependent model was trained twice, one with data augmentation and one without, identified by the suffixes *wa* and *woa*, respectively. When testing models, each sample would be predicted both in the order (*Drug A*, *Drug B*, *Cell line*) and in the order (*Drug B*, *Drug A*, *Cell line*). The average of the two predictions will be taken as the actual prediction. As results, the MSEs of CGMS, GraphSynergy-indep, and DeepDDS-indep are all lower than their order-dependent variants (Supplementary Table S3). The results also verify the effectiveness of data augmentation (see Supplementary Fig. S1), but even with data augmentation, there are still differences between predictions of the same sample with different input orders. We draw the kernel density estimation (KDE) plot on absolute errors between ground truth and predictions given by CGMS and its variants in Fig. 3. It can be seen that the errors of CGMS are more concentrated in the interval 0–10 than its variants. The same phenomenon can also be observed in GraphSynergy and DeepDDS (Supplementary Figs S2 and S3). The experiment findings demonstrate that the order-independent methods are capable of modeling the relations between drug combinations and cell lines more reasonably and more precisely.

### 3.4 Distribution of the whole-graph embeddings

**Figure 4:**
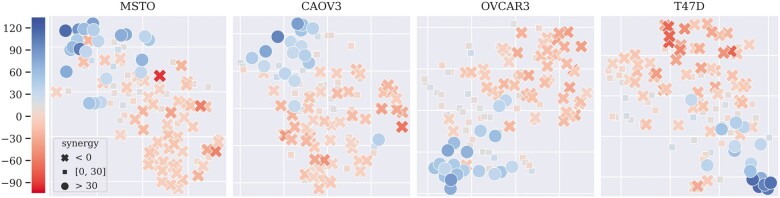
T-SNE results of four cell lines’ whole-graph embeddings distribution generated by CGMS. Dots in blue are samples whose synergy scores are higher than 30, squares with light color are samples whose synergy scores are in range [0, 30], and the crosses in orange are samples whose synergy scores are lower than 0.

CGMS generates the whole-graph embedding to reflect the interaction between a drug combination and a cell line. To explore how well the whole-graph embeddings can represent the samples, we visualized the whole-graph embeddings of four cell lines with t-SNE (Van der Maaten and Hinton 2008). The selected cell lines are those who occur most times in the strong-synergistic samples whose synergy scores are higher than 30 (Preuer *et al.* 2018). Figure 4 presents the distribution of t-SNE results of samples in test fold 1. The results in other test folds are plotted in Supplementary Figure S4. As shown in the figures, samples with high positive synergy scores (blue dots) and samples with negative scores (orange crosses) are separated clearly, which indicates that CGMS obtains a discriminative whole-graph embedding through HAN and can distinguish synergistic drug combinations for a certain cell line.

### 3.5 Analysis of graph attention in CGMS

**Figure 5:**
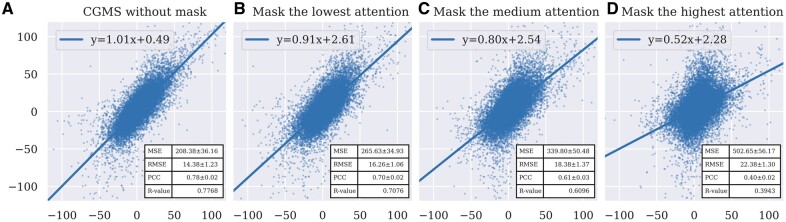
Relations between the predicted results and the ground truth. The values in *x*-axis are predicted scores, and the values in *y*-axis are the ground truth. The blue straight lines are fitted via the least squares method with *P*-value <1e−5. (A) The original results of CGMS without mask. (B) The results of masking the lowest attention. (C) The results of masking the medium attention. (D) The results of masking the highest attention.

The CGMS model introduces the graph attention mechanism to focus on the significant information for determining the synergism of drugs combinations. To analyze the rationality of the attention weights that CGMS has assigned for drugs and cell lines, we masked different parts of features according to the attention to observe the impact on the prediction. Specifically, we first extracted the attention values given by different heads in the last HAN layer. Then we masked the lowest, medium, and highest attention to zero to generate defective graph embeddings for making predictions, respectively. We calculated the evaluation metrics and visualized the relations between the predicted results and the ground truth with the least squares method. Figure 5 presents that the MSE and RMSE increase as we mask higher attentions. Meanwhile, the PCC and *R*-value between the predictions and the ground truth decrease. This phenomenon indicates that CGMS has learnt to pay more attention to more important parts for predicting the synergism of drug combinations on a certain cell line.

### 3.6 Ablation study for multi-task learning

CGMS adopts a multi-task learning technology to train the model for better generalization. To explore the effect of multi-task learning on the generalization ability of the model, we conducted ablation experiments. As mentioned before, we choose the synergy prediction task and the sensitivity prediction task for training CGMS. In the ablation study, we constructed two variants of CGMS, named CGMSyn and CGMSen, and compared them with CGMS on the two tasks, respectively. Each variant has the identical main structure with CGMS, but keeps only one MLP for prediction and is trained only on a single task.

Table 2 summarizes the results of the ablation study. The prediction error of CGMS is reduced compared to both variants trained only on a single task. On the synergy prediction task, CGMS achieves a 4.8% lower MSE than CGMSyn with a higher PCC. Besides, CGMS achieves a 11.7% lower MSE than CGMSen on the sensitivity prediction task, and increases the PCC by 0.05. It can be seen from the results that multi-task learning enhances the generalization ability of the CGMS model on both tasks.

**Table 2.** Experimental results of the ablation study.

	Synergy prediction		Sensitivity prediction	
Model	MSE	PCC	MSE	PCC
CGMS	**208.38 **±** 36.16**	**0.78 **±** 0.02**	**349.94 **±** 73.44**	**0.62 **±** 0.04**
CGMSyn	218.90 ± 33.83	0.75 ± 0.01		
CGMSen			396.41 ± 85.18	0.57 ± 0.05

Values of MSE and PCC are mean values ± 1 standard deviation. The best results are shown in bold.

## 4 Conclusion

We propose a complete graph-based approach for predicting anti-cancer synergistic drug combinations named CGMS. CGMS innovatively models the relationship between drug combinations and cell lines as a heterogeneous complete graph, and generates the whole-graph embedding with HAN for making stable, order-independent predictions. We adopt a multi-task learning technique to train CGMS to improve its generalization ability. We compare CGMS with six state-of-the-art methods on a public dataset in terms of predicting synergy scores for novel drug combinations, novel drugs, or novel cell lines. The results show that CGMS has significantly lower prediction errors than other compared methods in all scenarios. Further experiment results also present the benefit of eliminating the order dependency and the discrimination power of the whole-graph embeddings, interpret the rationality of attention mechanism and verify the contribution of multi-task learning to better generalization ability.

We also recognize that CGMS still has potential for further improvement. In this work, we focus more on the construction of deep model, while constructing the drugs’ and cell lines’ features following common practice. There are researches who have explored the way to integrate more informative features for synergistic drug combination prediction (Yang *et al.* 2021; Wang *et al.* 2022a,b), which inspire us to design input features of CGMS better.

Based on CGMS’s superior performance, its predicted scores can provide a reference for drug pre-screening. The drug combinations with higher predicted synergy scores may be more worthy of being tested. In conclusion, CGMS outperforms the state-of-the-art methods in predicting the synergy scores of drug combinations, which we anticipate will further advance the practical application of anti-cancer synergistic drug combination prediction models.

## Supplementary Material

btad351_Supplementary_Data

## Data Availability

The data and source code underlying this article are available in https://github.com/TOJSSE-iData/CGMS.
