# Supplementary material for: A complete graph-based approach with multi-task learning for predicting synergistic drug combinations
Source: Bioinformatics. 2023 Jun 1;39(6):btad351. doi: 10.1093/bioinformatics/btad351 (PMC10256571; doi:10.1093/bioinformatics/btad351)
Supplement: btad351_Supplementary_Data [file btad351_supplementary_data.docx]

**A complete graph-based approach with multi-task learning for predicting synergistic drug combinations**

*——Supplementary Information——*

**Xiaowen Wang^1^, Hongming Zhu^1^, Danyi Chen^1^, Yongsheng Yu^4^, Qi Liu^2,3*^, and Qin Liu^1*^**

^1^School of Software Engineering, Tongji University, Shanghai, 201804, China

^2^Translational Medical Center for Stem Cell Therapy and Institute for Regenerative Medicine, Shanghai East Hospital,

^3^Bioinformatics Department, School of Life Sciences and Technology, Tongji University, Shanghai, 200092, China

^4^Tasly Biopharmaceuticals Co., Ltd.

# S1 Construction of the Synergy Dataset and the Sensitivity Dataset

The synergy dataset was constructed based on the large-scale combination screening synergy dataset published by O’Neil et al (2016). The dataset covers the testing results of 583 different drug combinations against 39 human cancer cell lines using a 4 × 4 dosing regimen. The cell growth rate of each sample was measured in quadruplicate relative to the control group after 48 hours. Based on the experiment results, Preuer et al. (2018) calculated the Loewe synergy score corresponding to each sample. We further calculated the average value of Loewe scores corresponding to repeated samples, and finally obtained 22,737 samples consisting of drug combinations, cell lines, and synergy scores.

The sensitivity dataset was constructed based on DrugComb (Zagidullin et al., 2019). We first found all records containing the synergy score of drug pairs and the Relative Inhibition (RI) values of the two drugs on the cell line. We then flattened the records to samples in the format of (*Drug*, *Cell line*, *RI value*). Afterwards, we discarded records whose cell line's transcript per million (TPM) data could not be found in the Cancer Cell Line Encyclopedia (CCLE) dataset and are not contained in the O'Neil dataset. We also discarded records whose drug features could not be generated according to their simplified molecular input line entry system (SMILES) expressions via RDKit. To reduce data noise, we only retained the samples where the drug is tested on no less than 5 cell lines. Finally, we constructed a sensitivity dataset of 11,766 samples with 346 drugs and 175 cell lines.

CGMS is evaluated in three different scenarios, that are the leave-drug combination-out scenario, the leave-cell line-out scenario, and the leave-drug-out scenario. In different scenarios, the two datasets were divided into folds differently to perform 5-fold cross-validation.

In the leave-drug combination-out scenario, the synergy dataset was divided into 5 folds in terms of the drug combination, following Preuer et al. (2018). Since each drug combination will only appear in one fold, each of the five folds could be used as a test fold. It should be noted that in this scenario, all drugs and cell lines occur in each fold. The sensitivity dataset was sorted by drug name, and the sorted samples were put into each fold in turn. Since each drug was tested on at least 5 cell lines, the drugs are guaranteed to appear in each fold.

In the leave-cell out-scenario, similar to the leave-drug combination-out scenario, the synergy dataset was divided into 5 folds evenly in terms of cell lines. For the sensitivity dataset, we first put samples whose cell line is involved in the O'Neil dataset into the same fold as the synergy dataset and then put the remaining samples into 5 folds in terms of cell lines.

In the leave-drug-out scenario, we first divided all drugs in the synergy dataset into two sets. The first set of drugs is named the exhaustive set, where the drugs were tested together with 37 other drugs. The other drugs were put into the second set, called supplemental set. For each test fold, we first picked 2 drugs from the exhaustive set as well as 2 drugs from the supplemental set and then selected samples that contain one of the 4 drugs to form the test fold. The remaining samples were randomly divided into 4 folds for training. Therefore, we constructed 5 pairs of training/test synergy datasets, and 20 drugs were used for testing in the whole 5-fold cross-validation procedure. For the sensitivity dataset, we first arranged samples containing the 20 test drugs into the same fold as the synergy dataset, and then put the remaining samples into 5 folds evenly in terms of drugs.

# S2 Encoding drug features and cell line features with Auto-Encoder

We first constructed the original feature matrices for drugs and cell lines, respectively. We find that the drug and cell line features are high-dimensional and sparse, and taking such data directly as input will cause the model to have a large number of parameters. Since the scale of existing synergistic drug combination experiments is still insufficient, a larger parameter size means that the model has a higher risk of overfitting and higher training overhead. Therefore, we first used Auto-Encoders to perform feature extraction on the raw features of drugs and cell lines to obtain low-dimensional, dense feature representations for drugs and cell lines.

The encoder and decoder of the Auto-Encoder used in this paper are a set of Multi-layer Perceptrons (MLPs) whose structures are symmetric. We note the input of encoder as $\mathbf{x}$, the encoded latent variable as $\mathbf{z}$, and the re-constructed vector decoded by the decoder as $\hat{\mathbf{x}}$. The training object of the Auto-Encoder is to minimize the reconstruction error $\mathcal{L}_{re-con}$:

$$\begin{aligned} \mathbf{z}= \mathrm{FC}_{2}\left( \mathrm{Dropout}\left( \mathrm{ReLU}\left( \mathrm{FC}_{1}\left( \mathbf{x} \right) \right), 0.1 \right) \right) \\ \hat{\mathbf{x}} = \mathrm{FC}_{4}\left( \mathrm{Dropout}\left( \mathrm{ReLU}\left( \mathrm{FC}_{3}\left( \mathbf{z} \right) \right), 0.1 \right) \right) \\ \mathcal{L}_{re-con}\boldsymbol{&=} \left\| \hat{\mathbf{x}}-\mathbf{x} \right\|_{2} \end{aligned}$$

where $\mathrm{FC}_{i}$, $i\in\left\{ 1..4 \right\}$ is the fully-connected (FC) layer, ReLU is the Rectified Linear Unit. For the drug feature Auto-Encoder, the dimension of the input feature $\mathbf{x}_{d}$ is 1213. For the cell line feature Auto-Encoder, the dimension of the input feature $\mathbf{x}_{c}$ is 5001. The dimension of latent variables $\mathbf{z}_{d}$ and $\mathbf{z}_{c}$ is 256. We trained both Auto-Encoders using the AdamW optimizer with a learning rate of 0.001. Since the dimension of $\mathbf{x}_{c}$ is higher than that of $\mathbf{x}_{d}$, we set the number of epochs to 100 for the cell line feature Auto-Encoder and 50 for the drug feature Auto-Encoder. The extracted 256-dimensional feature vectors for both drugs and cell lines are used as feature vectors for each node in the complete graph.

# S3 Evaluation Details of Baseline Methods in Method Comparison

CGMS is compared with six advanced methods on the regression task with the widely used O’Neil dataset in three different scenarios. In the leave-drug combination-out scenario, the results of DeepSynergy, AuDNNSynergy, PRODeepSyn and TranSynergy come from the original papers, since all the four methods have been evaluated with the O’Neil dataset by their authors, respectively. The experiment results of EC-DFR and DeepDDS were obtained by re-implementing the methods. In the leave-cell line-out and leave-drug-out scenarios, all the results of baseline methods except AuDNNSynergy were obtained by re-running their codes. We do not report the results of AuDNNSynergy in these two scenarios since they do not provide their feature data and code of implementation. All the methods were evaluated with the same dataset according to the scenario as much as possible, as some methods were not feasible for certain drugs or cell lines. The detailed implementation is as follows:

- **PRODeepSyn.** We re-ran the source code of PRODeepSyn with the synergy dataset in the leave-cell line-out scenario and the leave-drug-out scenario. We selected the number of hidden units in the first fully-connected (FC) layer from {2048, 4096, 8192}, and the learning rate from {0.001, 0.0001, 0.00001} as the original paper did. We kept other settings the same as the original paper.
- **TranSynergy.** We re-ran the source code of TranSynergy with the synergy dataset in the leave-cell line-out scenario and the leave-drug-out scenario. We excluded samples related to 2 drugs and 4 cell lines because TranSynergy could not generate features for these drugs and cell lines as described in its paper. The results were obtained following the settings and best hyperparameters given in its paper.
- **DeepSynergy.** We re-implemented DeepSynergy using PyTorch 1.10. We first evaluated DeepSynergy in the leave-drug combination-out scenario with the same settings as its paper to verify the correction of our implementation. The mean square error (MSE) is 251.13, which is nearly the same as the result given by DeepSynergy (255.49). We then evaluated DeepSynergy with the synergy dataset in the leave-cell line-out scenario and the leave-drug-out scenario. We found that DeepSynergy was easy to overfit in the leave-cell line-out and leave-drug-out scenario with settings in its paper. After times of attempt, we fixed the learning rate of DeepSynergy to 0.001, and the number of hidden units from {[1024, 1024], [1024, 512], [512, 512], [512, 256]}. We set the batch size to 512 and optimized the model with Adam optimizer. The unmentioned settings were kept the same as its paper.
- **DeepDDS.** We re-ran the source code of DeepDDS to carry out evaluation using the DeepDDS-GAT model on the regression task. DeepDDS was also evaluated on a subset of datasets in different scenarios, where 2 drugs and 8 cell lines were removed due to the data availability. In the leave-drug combination-out scenario, we selected the learning rate from {0.01, 0.005, 0.001, 0.0005} and set the batch size to 128. We set the number of attention heads of the Graph Attention Network (GAT) to 10 and the GAT hidden units to [256, 256]. We selected the number of hidden layers of the MLP from {1, 2, 3} and selected the number of hidden units from {1024, 2048, 4096}. Other hyperparameters were set following the best settings in the original paper. In the leave-cell line-out scenario and the leave-drug-out scenario, we selected the GAT hidden units from {[256, 256], [1024, 512]}, the FC hidden units from {[1024, 512, 128], [1024, 512], [4096], [1024]} according to the paper's setting and our attempt. We set other hyperparameters following the optimal value given by DeepDDS.
- **EC-DFR.** We re-ran the source code of EC-DFR to carry out the evaluation. The input features of drugs and cell lines were the same encoded features as CGMS. We selected the error rate from {20%, 30%, 60%} and set the resample times to 2. We set the number of XGBoost models in each ‘layer’ of EC-DFR to 6, and set the max number of trees in each XGBoost to 200. We selected the learning rate of XGBoost from {1, 0.3, 0.1, 0.05} and kept other hyperparameters of XGBoost as default. The max depth of EC-DFR was set to 4.

Since the results of the five methods are order-dependent, we doubled the samples for training by exchanging the input drug order, and toke the average of predictions with both orders as their final predictions.

The results of DeepDDS and EC-DFR are less competitive. We think this may be related to the fact that DeepDDS is more suitable for simple classification tasks, while EC-DFR is oriented towards small datasets.

# S4 Early Stopping with 5-fold Nested Cross-Validation

CGMS is evaluated on the O’Neil dataset with 5-fold cross-validation. To avoid overfitting, we also applied the early stopping strategy during model training. The detailed steps are as follows:

1. Split the synergy dataset and the sensitivity dataset into five folds according to the test scenario.
2. Choose one fold as a test fold each time and note the other four folds as outer training folds. For example, choose the first fold of the synergy dataset and the first fold of the sensitivity dataset as the test fold and the other folds as outer training folds.
3. Select a set of hyperparameters for evaluation.
4. For each fold in outer training folds, choose one fold of the two datasets respectively as validation folds each time and note the other folds as inner training folds.
5. Train the model on the inner folds with the selected hyperparameter set and keep watching the MSE over the validation folds. Stop training when the MSE on the validation fold from the synergy dataset has not decreased for specified epochs, 50 for example.
6. Repeat Step 4-5 until each outer training fold from the synergy dataset has been chosen as the validation fold. Calculate the mean MSE on the four outer training folds as the performance of the selected hyperparameters.
7. Repeat Step 3-6 to evaluate all hyperparameter sets and select the hyperparameter set with the best performance.
8. Randomly split the outer training folds from the synergy dataset to 10 folds. Randomly choose one fold as a small validation fold for the early stopping strategy and train the model with the other 9 folds. Train CGMS with the selected hyperparameter set in Step 7. Note that here we do not split the sensitivity dataset into folds since we do not monitor the volatility of MSE on folds from the sensitivity dataset for early stopping.
9. Evaluate the trained model from Step 8 on the test fold selected in Step 2.
10. Repeat Step 2-9 to complete the 5-fold nested cross-validation with early stopping.

# S5 Detailed Results of Compared Methods in Different Scenarios

In this section, we report the evaluation results of CGMS and other baseline methods in the leave-cell line-out scenario and in the leave-drug-out scenario. The reported metrics include the mean square error (MSE), the root mean square error (RMSE), the confidence interval, and the Pearson correlation coefficient (PCC).

| **Method** | **MSE** | **RMSE** | **Confidence Interval 95%** | **PCC** |
| --- | --- | --- | --- | --- |
| CGMS | **386.73 ± 152.21** | **19.34 ± 3.59** | **[197.76, 575.70]** | **0.53 ± 0.05** |
| TranSynergy | 415.77 ± 131.71 | 20.16 ± 3.08 | [252.25, 579.28] | 0.50 ± 0.05 |
| PRODeepSyn | 417.70 ± 168.85 | 20.07 ± 3.83 | [208.08, 627.32] | 0.48 ± 0.05 |
| DeepSynergy | 431.04 ± 156.84 | 20.45 ± 3.57 | [236.33, 625.75] | 0.51 ± 0.06 |
| EC-DFR | 509.50 ± 239.28 | 22.04 ± 4.88 | [212.44, 806.56] | 0.43 ± 0.05 |
| DeepDDS | 528.82 ± 274.13 | 22.37 ± 5.32 | [188.50, 869.14] | 0.37 ± 0.04 |

Table S1: Results of method comparison in the leave-cell line-out scenario.

| **Method** | **MSE** | **RMSE** | **Confidence Interval 95%** | **PCC** |
| --- | --- | --- | --- | --- |
| CGMS | **319.28 ± 56.07** | **17.80 ± 1.58** | **[249.67, 388.89]** | **0.62 ± 0.05** |
| PRODeepSyn | 442.87 ± 90.09 | 20.94 ± 2.11 | [331.03, 554.72] | 0.42 ± 0.06 |
| DeepSynergy | 456.85 ± 99.80 | 21.25 ± 2.28 | [332.95, 580.75] | 0.41 ± 0.04 |
| TranSynergy | 466.77 ± 68.37 | 21.54 ± 1.66 | [381.89, 551.65] | 0.46 ± 0.04 |
| EC-DFR | 489.25 ± 90.35 | 22.03 ± 2.02 | [377.08, 601.42] | 0.43 ± 0.02 |
| DeepDDS | 518.08 ± 102.57 | 22.65 ± 2.24 | [390.75, 645.41] | 0.30 ± 0.04 |

Table S2: Results of method comparison in the leave-drug-out scenario

# S6 Details of Predictions with Different Input Orders of Drug pairs

We implemented the order-dependent variants of CGMS, CGMS-ord, as well as the order-independent variants of GraphSynergy and DeepDDS. The CGMS-ord model was implemented by changing the input of CGMS's MLPs from the whole-graph embeddings to the concatenation of node embeddings given by the second Heterogeneous Graph Attention Network (HAN) layer. The GraphSynergy-indep and DeepDDS-indep were implemented by adding the self-attention mechanism to the origin model. Specifically, for GraphSynergy, the final representation vectors of drugs and cell lines are first projected by entity type-related matrices into a unified space, respectively. The projected vectors are aggregated with multi-head self-attention mechanism to represent the combination of two drugs and one cell line. The aggregated representation is then fed into a MLP with one hidden layer to predict the therapy score. Similarly, for DeepDDS, the embedding vectors of drugs and cell line are also aggregated with multi-head self-attention mechanism instead of using the sequential concatenation. It should be noted that we also use a MLP with one hidden layer to replace the Transformation matrix in GraphSynergy for a fair comparison.

Each order-dependent model was trained twice, once with data augmentation and once without. CGMS-ord-wa and CGMS-ord-woa was trained using the same settings as CGMS. For GraphSynergy, we selected the batch size from {256, 512}, the training epoch from {10, 20, 50}, the dimension of embeddings from {16, 32, 64}. We set the learning rate to 0.001, the depth of the interaction field to 2, the regularizer weights $\lambda_{1}$ and $\lambda_{2}$ to ${10}^{-5}$ and ${10}^{-6}$, referring to the settings of GraphSynergy. We set the sample size of neighbors in each layer to 256. We set the number of attention heads in GraphSynergy-indep to 8 as CGMS, the activate function of MLP hidden layer to ReLU, and the dropout rate to 0.5. We first trained GraphSynergy with the data augmentation strategy (GraphSynergy-wa) in the leave-drug combination-out scenario using five-fold cross-validation. The hyperparameters of the optimal results were used to train GraphSynergy-indep and GraphSynergy-woa. For DeepDDS-indep and DeepDDS-woa, their hyperparameters kept the same as DeepDDS's optimal settings in the leave-drug combination-out scenario. The number of attention heads in DeepDDS-indep was set to 10 according to its original paper.

The experiment results are shown in Supplementary Table S3. We also visualized the kernel density estimation (KDE) on absolute differences between the predictions of the same sample with different input orders for CGMS-ord-wa and CGMS-ord-woa in Figure S3, which shows the effect of data augmentation on reducing the difference caused by changing the input order of drugs.

| **Method** | **MSE** | **RMSE** |
| --- | --- | --- |
| **CGMS** | **208.38 ± 36.16** | **14.38 ± 1.23** |
| CGMS-ord-wa | 223.49 ± 25.76 | 14.93 ± 0.85 |
| CGMS-ord-woa | 252.17 ± 29.07 | 15.85 ± 0.90 |
| GraphSynergy-indep | 414.93 ± 53.21 | 20.33 ± 1.29 |
| GraphSynergy-wa | 426.13 ± 46.22 | 20.61 ± 1.10 |
| GraphSynergy-woa | 452.66 ± 67.27 | 21.22 ± 1.59 |
| DeepDDS-indep | 261.79 ± 37.64 | 16.14 ± 1.16 |
| DeepDDS-wa (DeepDDS) | 271.62 ± 30.15 | 16.45 ± 0.93 |
| DeepDDS-woa | 314.96 ± 23.50 | 17.73 ± 0.67 |

Table S3: Results of CGMS, GraphSynergy, DeepDDS, and their variants.


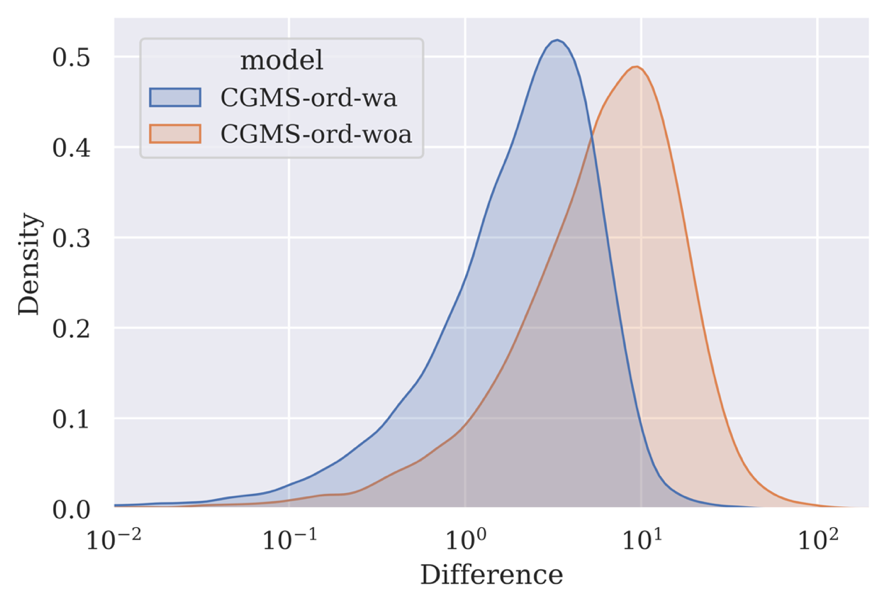


Figure S1. KDE plot on difference between predictions for the same sample with different drug input orders, where the difference is absolute value.


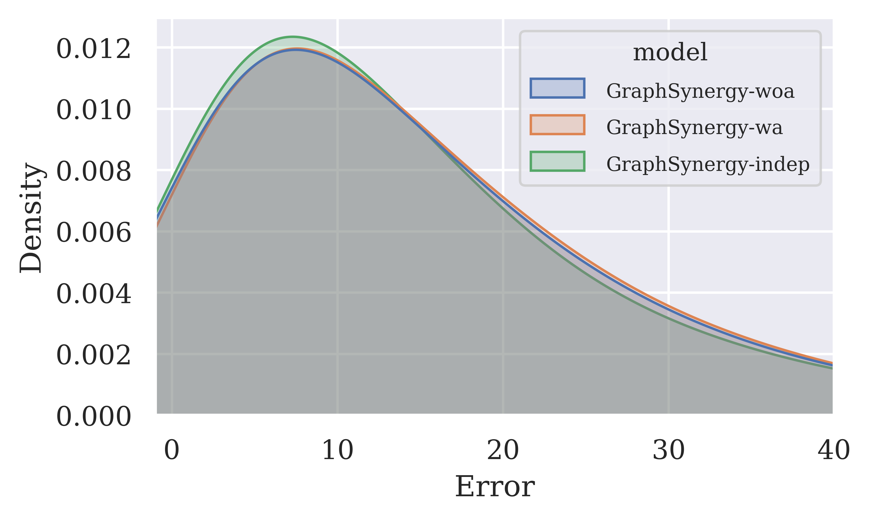


Figure S2: KDE plot on absolute errors between the predictions and the ground truth of GraphSynergy and its variants.


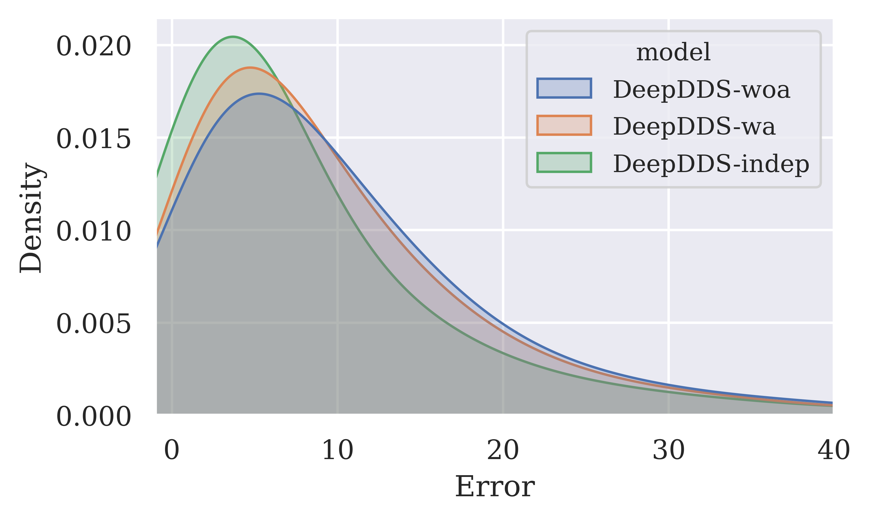


Figure S3: KDE plot on absolute errors between the predictions and the ground truth of DeepDDS and its variants.

# S7 Distribution of the Whole-graph Embeddings

We visualized the whole-graph embeddings of four cell lines generated by CGMS with t-SNE. The selected cell lines are those who occur most times in the strong-synergistic samples whose synergy scores are higher than 30. All of these cell lines are involved in no less than 8 strong-synergistic samples in each fold. As shown in Figure S2, samples with high positive synergy scores (marked with blue dots) and samples with negative scores (marked with orange crosses) are separated clearly.


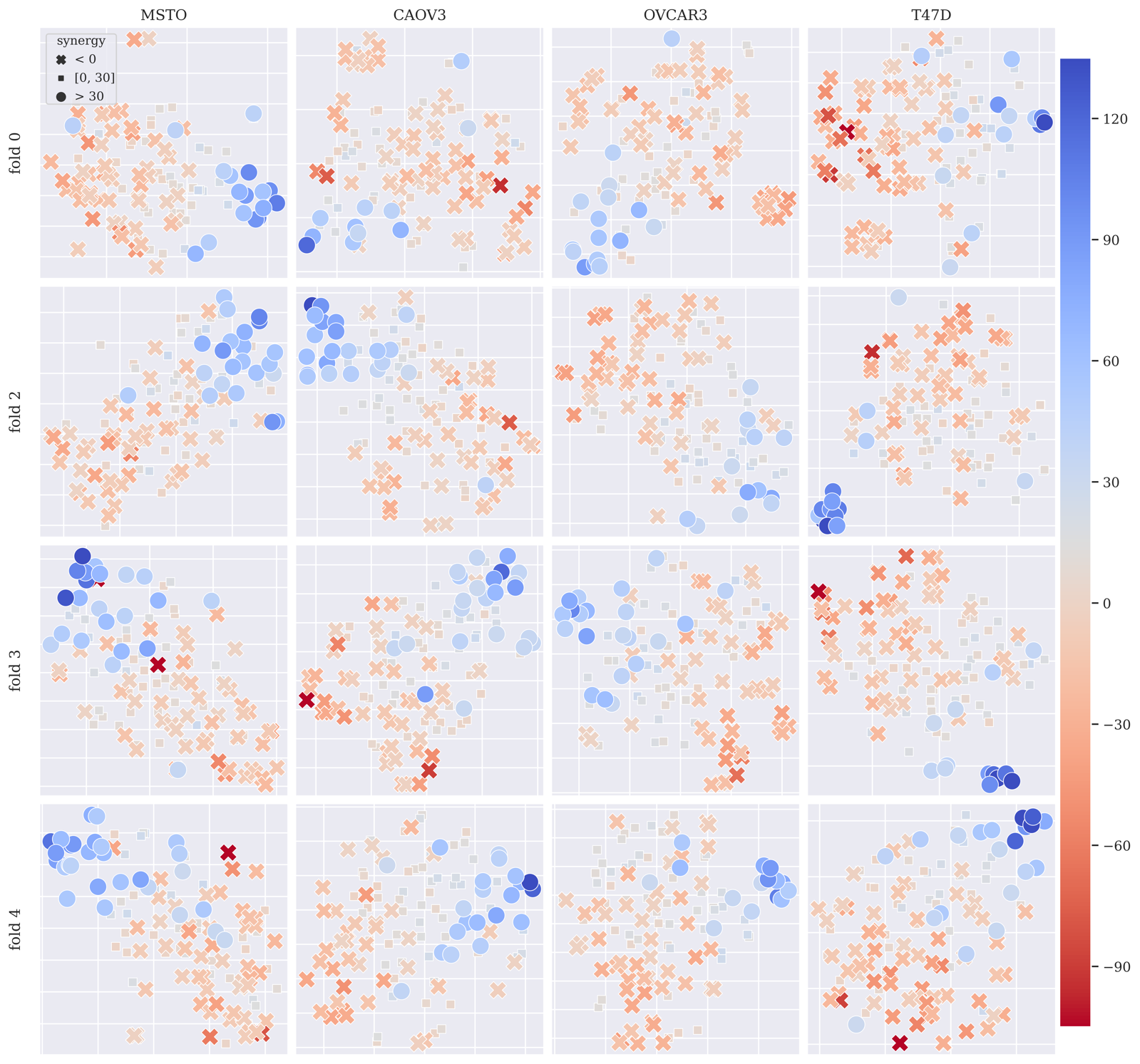


Figure S4. T-SNE results of four cell lines' whole-graph embeddings distribution generated by CGMS. Dots in blue are samples whose synergy scores are higher than 30, squares with light color are samples whose synergy scores are in range [0, 30], and the crosses in orange are samples whose synergy scores are lower than 0.

# References

1. O'Neil, Jennifer et al. “An Unbiased Oncology Compound Screen to Identify Novel Combination Strategies.” Molecular cancer therapeutics vol. 15,6 (2016): 1155-62.
2. Preuer, Kristina et al. “DeepSynergy: predicting anti-cancer drug synergy with Deep Learning.” Bioinformatics (Oxford, England) vol. 34,9 (2018): 1538-1546.
3. Zagidullin, Bulat et al. “DrugComb: an integrative cancer drug combination data portal.” Nucleic acids research vol. 47,W1 (2019): W43-W51.
